# Concurrent nanoscale surface etching and SnO_2_ loading of carbon fibers for vanadium ion redox enhancement

**DOI:** 10.3762/bjnano.10.99

**Published:** 2019-04-30

**Authors:** Jun Maruyama, Shohei Maruyama, Tomoko Fukuhara, Toru Nagaoka, Kei Hanafusa

**Affiliations:** 1 Research Division of Environmental Technology, Osaka Research Institute of Industrial Science and Technology, 1-6-50, Morinomiya, Joto-ku, Osaka 536-8553, Japan; 2 Research Division of Materials Science and Engineering, Osaka Research Institute of Industrial Science and Technology, 1-6-50, Morinomiya, Joto-ku, Osaka 536-8553, Japan; 3 Power Systems R&D Center, Sumitomo Electric Industries, 1-1-3, Shimaya, Konohana-ku, Osaka 554-0024, Japan

**Keywords:** carbon fiber, electrode reactions, metal-oxide nanoparticles, redox flow batteries, surface etching

## Abstract

Facile and efficient methods to prepare active electrodes for redox reactions of electrolyte ions are required to produce efficient and low-cost redox flow batteries (RFBs). Carbon-fiber electrodes are widely used in various types of RFBs and surface oxidation is commonly performed to enhance the redox reactions, although it is not necessarily efficient. Quite recently, a technique for nanoscale and uniform surface etching of the carbon fiber surface was developed and a significant enhancement of the negative electrode reaction of vanadium redox flow batteries was attained, although the enhancement was limited to the positive electrode reaction. In this study, we attempted to obtain an additional enhancement effect of metal-oxide nanoparticles without the need for further processing steps. A coating with carbonaceous thin films was obtained coating by sublimation, deposition, and pyrolysis of tin(II) phthalocyanine (SnPc) on a carbon fiber surface in a single heat-treatment step. The subsequent thermal oxidation concurrently achieved nanoscale surface etching and loading with SnO_2_ nanoparticles. The nanoscale-etched and SnO_2_-loaded surface was characterized by field-emission scanning electron microscopy (FESEM), Raman spectroscopy, and X-ray photoelectron spectroscopy (XPS). The activity for the vanadium ion redox reactions was evaluated by cyclic voltammetry (CV) to demonstrate the enhancement of both the positive and negative electrode reactions. A full cell test of the vanadium redox flow battery (VRFB) showed a significant decrease of the overpotential and a stable cycling performance. A facile and efficient technique based on the nanoscale processing of the carbon fiber surface was presented to substantially enhance the activity for the redox reactions in redox flow batteries.

## Introduction

Redox flow batteries (RFBs) are energy conversion and storage devices that involve the reduction and oxidation of electroactive species in electrolyte solutions and have attracted much attention due to their scalability and safety. Various types of RFBs have been developed using aqueous and nonaqueous electrolytes with inorganic and organic electroactive species [1–4]. There is an increasing demand for electrodes that are active in the redox reactions of every type of RFBs to enhance the reaction rate, improve the energy efficiency [5–6], and to allow for a compact cell design. Feasible production methods are also required to provide low production cost.

Carbon-fiber electrodes are conventionally used in RFBs and surface oxidation is often performed to enhance the redox reactions [7–14], although a sufficient activity has not yet been obtained. Recently, we found a method to efficiently expose the edge planes of the carbon fiber surface by nanoscale etching, which had a significant enhancement effect on the redox reactions of vanadium ions [15]. The reactions shown below are involved in the vanadium redox flow batteries (VRFBs), which are in the most advanced stage of research and development:


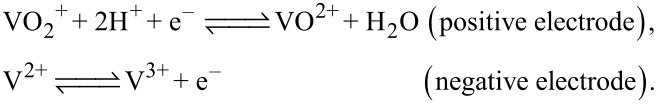


Nanoscale surface etching was attained by coating the surface with a carbonaceous thin film derived from cobalt(II) phthalocyanine (CoPc) and subsequent thermal oxidation followed by acid washing. The carbonaceous thin film was formed by sublimation, deposition, and pyrolysis of CoPc on the carbon fiber surface during a single heat-treatment step using a conventional crucible. The treatment substantially enriched edge planes and produced an enhanced activity for the positive and negative electrode reactions, although the enhancement of the former was limited.

It has been recognized that the modification of the carbon fiber surface by metal oxide nanoparticles also enhances the redox reactions [16–18]. In this study, we attempted the combination of the effects of edge-plane exposure and loading with metal-oxide nanoparticles to further enhance the activity and found that through the thermal oxidation of the carbonaceous thin film derived from SnPc both types of enhancement can be concurrently achieved. The formed metal oxide, SnO_2_, is one of the candidates for a durable catalyst support used in an acidic electrolyte [19]; thus, is assumed to also be stable in the RFB environment. The activity for both the positive and negative electrode reactions of a VRFB were clearly enhanced at the finely etched and SnO_2_-loaded carbon-fiber electrode and a stable performance was demonstrated by full cell cycle tests.

## Results and Discussion

### Concurrent surface etching and SnO_2_ loading

Graphitic carbon paper (TGP-H-090, Toray, abbreviated as TGP) was used as the substrate. The SnPc-derived carbonaceous thin film (CSnPc; obtained thorugh sublimation, deposition, and pyrolysis of SnPc in a single-step heat treatment in an Ar atmosphere at 700 °C) was coated on the carbon fiber surface following the method reported in [15,20]. The obtained sample was labeled TGP-CSnPc. Then, a heat treatment in air at *T* = 500, 550, 600, and 650 °C was performed to obtain TGP-CSnPc-*T*Air. The thermal oxidation at 550 °C was also performed without CSnPc for comparison. The treatment conditions and the obtained samples are summarized in Table 1.

**Table 1:** Conditions for TGP surface treatments and obtained samples.

	SnPc-derived carbonaceous thin film	thermal oxidation temperature [°C]

TGP	—	—
TGP-550Air	—	550
TGP-CSnPc	coated	—
TGP-CSnPc-500Air	coated	500
TGP-CSnPc-550Air	coated	550
TGP-CSnPc-600Air	coated	600
TGP-CSnPc-650Air	coated	650

### Surface morphology

**Figure 1:**
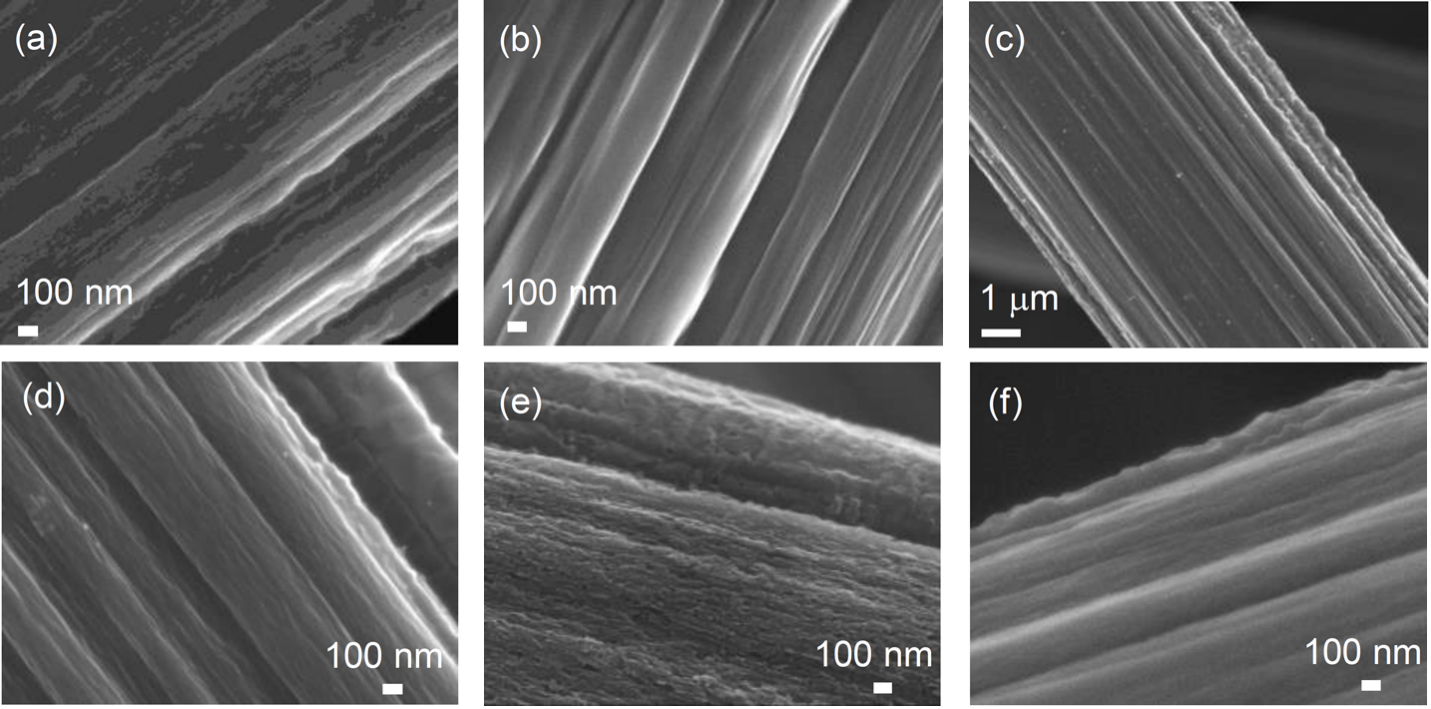
FESEM images of (a) TGP, (b) TGP-CSnPc, (c) TGP-CSnPc-550Air, (d) magnified view of (c), and (e) TGP-CSnPc-650Air. FESEM image of TGP-550Air (f) is also shown for comparison. FESEM images of TGP-CSnPc-*T*Air (*T* = 500, 550, 600 and 650 °C) are shown in Figure S2 (Supporting Information File 1).

The FESEM images of TGP, TGP-CSnPc, and TGP-CSnPc-550Air are shown in Figure 1 and Figure S1 (Supporting Information File 1). The surface morphology of the carbon fiber coated with CSnPc is similar to that without the coating. After the thermal oxidation of TGP-CSnPc at 550 °C, tin-oxide nanoparticles were generated on the surface. In addition, there are many shallow elongated dents along the fiber axis, which were generated by the fine surface etching. Although a clear demonstration of this surface-structure change is difficult through FESEM observation only, Raman spectroscopy and electrochemical measurements can show clear differences as described below.

The degree of the surface etching depends on the temperature of the thermal oxidation (Figure 1e and Figure S2, Supporting Information File 1). The surface was roughened with an increase in the temperature. It should be noted that the roughening was uniformly attained over the entire surface at every treatment temperature.

### Edge plane exposure

**Figure 2:**
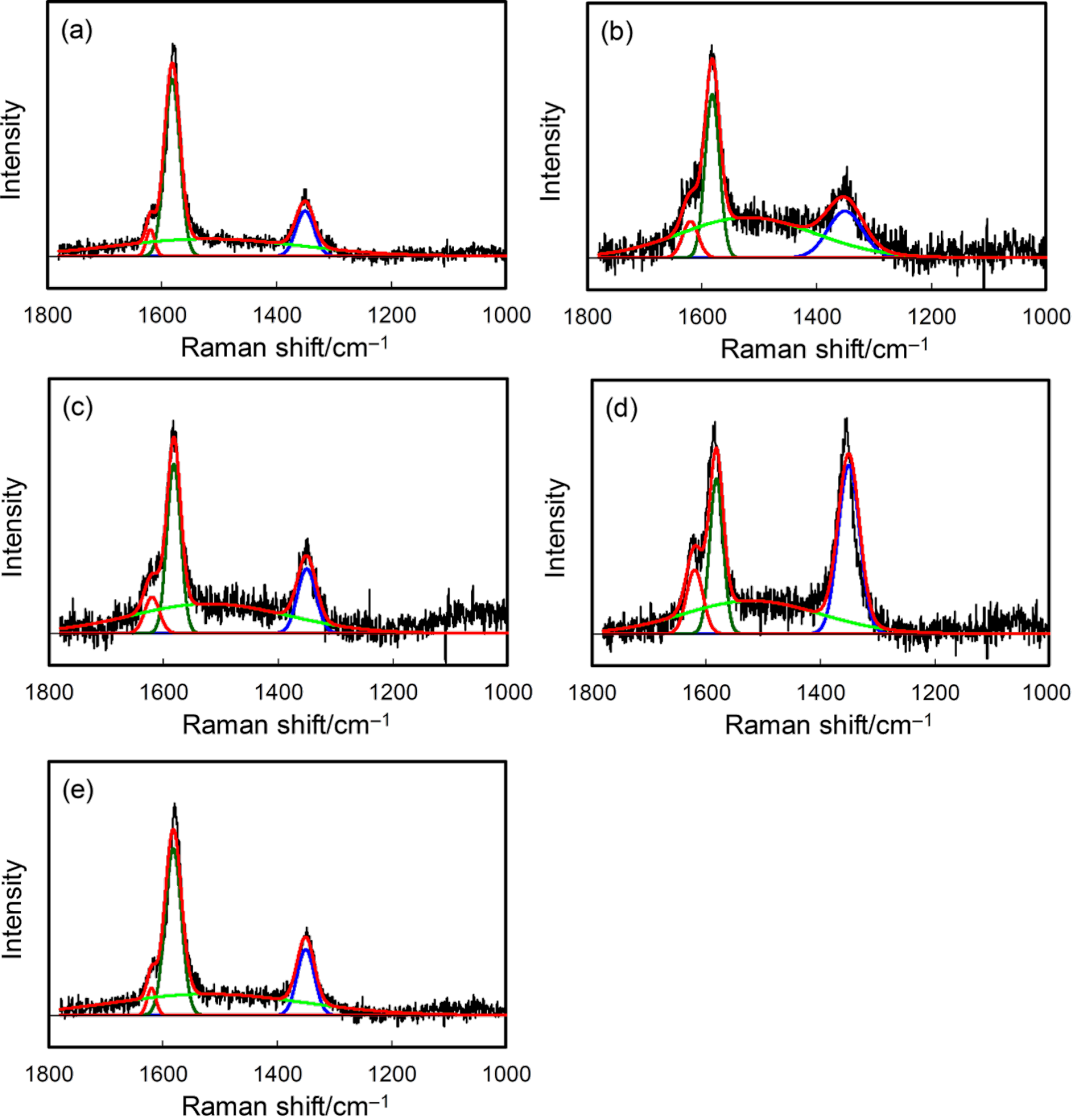
Raman spectra of (a) TGP, (b) TGP-CSnPc, (c) TGP-CSnPc-550Air, (d) TGP-CSnPc-650Air, and (e) TGP-550Air. The deconvoluted components, D2, G, Am, D, and the fitting result are shown in orange, green, light green, blue, and red lines, respectively. The spectra of TGP-CSnPc-*T*Air (*T* = 500, 550, 600 and 650 °C) are given in Figure S3 (Supporting Information File 1).

The further evaluation of the etched surface was carried out by Raman spectroscopy. Figure 2 shows the Raman spectra of TGP and the treated samples. After the coating of TGP with CSnPc, the Am and D2 peaks appeared in addition to the G and D peaks. The peaks are ascribed to amorphous carbon, the surface graphene layers as a disordered graphitic lattice, the ideal graphitic lattice, and the graphene layer edges also as the disordered graphitic lattice, respectively [21–22]. The presence of the Am peak indicates that CSnPc is amorphous. The Am peak is decreased (Table 2) and the D peak is increased in the spectrum for TGP-CSnPc-550Air. The ratios between the intensities of the D peak and the G peak (*I*_D_/*I*_G_) increased from 0.255 (TGP) to 0.382 (TGP-CSnPc-550Air), suggesting the exposure of the edge planes on the carbon fiber surface and also a slight retention of the amorphous carbon [23]. This assumption is based on the general recognition that the ratio is related to the concentration of the defects and the extent of the structural disorder [21]. The *I*_D_/*I*_G_ value is similar to that of TGP-550Air. The *I*_D_/*I*_G_ value depends on the thermal oxidation temperature and a highly developed D peak and a slight increase in the Am peak intensity were observed in the spectrum for TGP-CSnPc-650Air, which is in agreement with the FESEM image.

**Table 2:** Ratio between the intensity of the D, Am, and D2 peaks and that of the G peak.

	*I*_D_/*I*_G_	*I*_Am_/*I*_G_	*I*_D2_/*I*_G_

TGP	0.255	0.097	0.150
TGP-550Air	0.395	0.127	0.163
TGP-CSnPc	0.285	0.246	0.222
TGP-CSnPc-500Air	0.315	0.185	0.216
TGP-CSnPc-550Air	0.382	0.175	0.214
TGP-CSnPc-600Air	0.556	0.167	0.223
TGP-CSnPc-650Air	1.087	0.212	0.413

### Surface species

**Figure 3:**
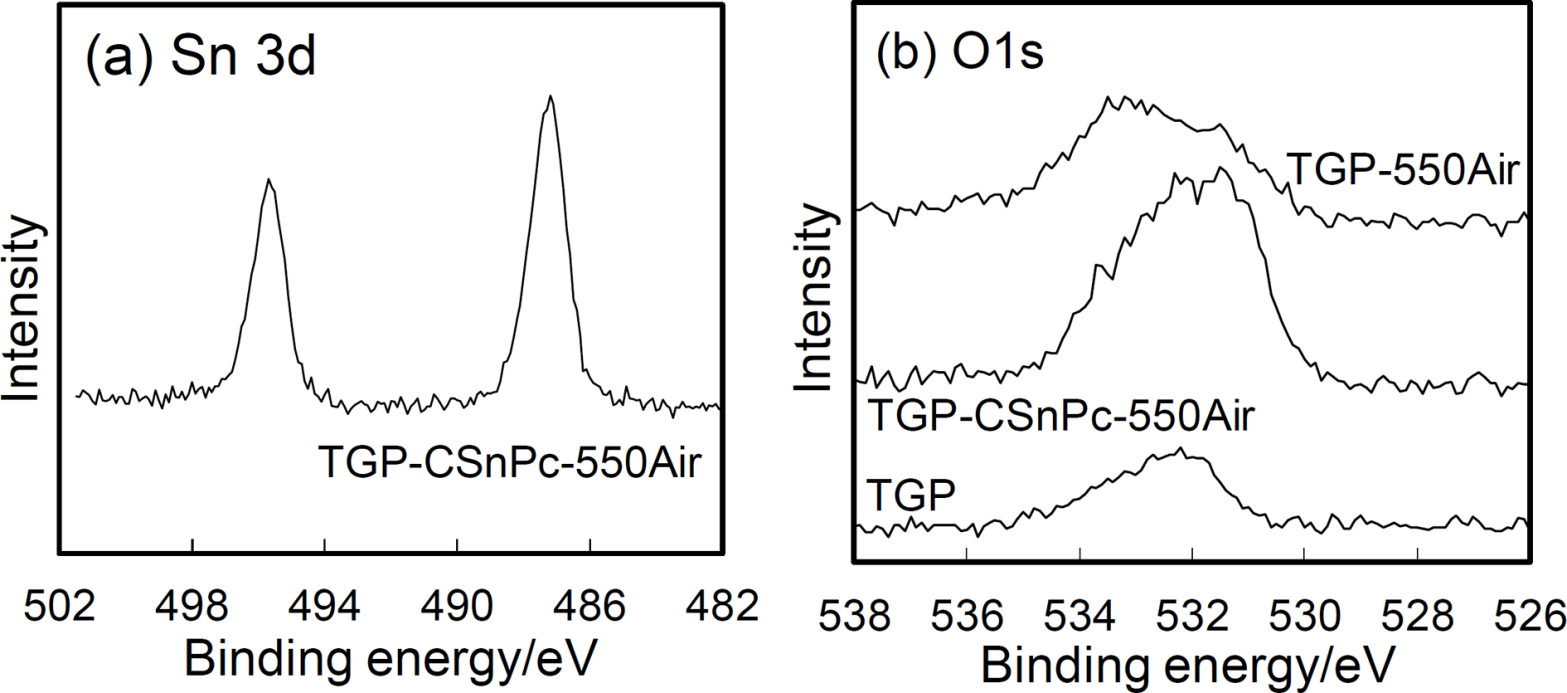
XPS spectra of (a) Sn 3d and (b) O 1s in TGP, TGP-CSnPc-550Air, and TGP-550Air.

The presence of tin oxide on the thermally oxidized surface of the CSnPc-coated carbon fibers was confirmed by XPS. It should be noted here that the Sn content was below the detection limit for the elemental mapping by energy-dispersive X-ray spectrometry because the SnO_2_ particles were of the order of nanometers and present only on the surface of the larger-scale carbon-fiber material. The XPS analysis area was 0.3 × 0.7 mm, yielding average values of the sample. Figure 3 shows the Sn 3d and O 1s XPS spectra of TGP, TGP-CSnPc-550Air, and TGP-550Air. The Sn 3d spectra indicated the presence of Sn in the form of SnO_2_ [24–26]. Although the C 1s spectra show little appreciable difference among these samples (Figure S4, Supporting Information File 1), the O 1s spectra for TGP-CSnPc-550Air clearly showed the presence of oxygen attributed to the metal oxide and oxygen-containing surface functional groups (Figure S5, Supporting Information File 1). The amount of the latter was comparable to that in TGP-550Air. Table 3 shows the surface compositions of these samples. The CSnPc coating and its conversion through thermal oxidation were reflected by the change in nitrogen surface concentration from TGP-CSnPc to TGP-CSnPc-550Air. The high oxygen surface concentration in TGP-CSnPc was attributed to its rough surface due to the structural disorder of the amorphous carbon, which was susceptible to oxidation upon exposure to air after the CSnPc deposition. The graphitic surface is much less susceptible to the oxidation [27]. Thus, the oxygen surface concentration decreases from TGP-CSnPc to TGP-CSnPc-550Air also suggested the removal of the CSnPc coating.

**Table 3:** Surface concentrations of C, O, and Sn [atom %].

	C 1s	O 1s	N 1s	Sn 3d

TGP	99.54	0.46	—	—
TGP-550Air	99.12	0.88	—	—
TGP-CSnPc	90.45	5.59	3.16	0.8
TGP-CSnPc-550Air	98.26	1.42	0.05	0.27

### Electrochemical behavior without vanadium ions

**Figure 4:**
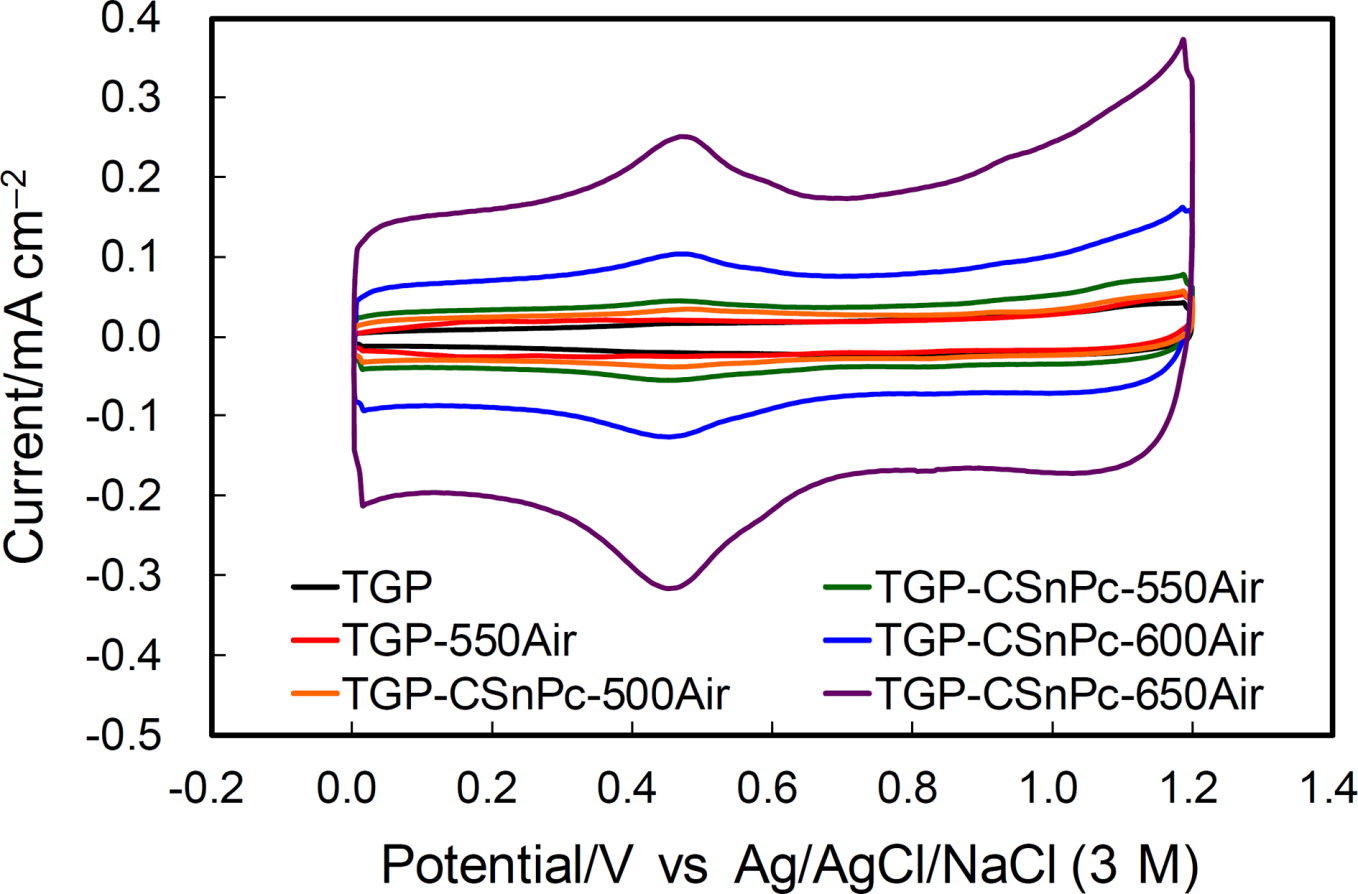
Cyclic voltammograms in Ar-saturated 2 M H_2_SO_4_ at 25 °C for TGP, TGP-550Air, and TGP-CSnPc-*T*Air (*T* = 500, 550, 600 and 650 °C). The reference electrode was Ag/AgCl/NaCl (3 M). The counter electrode was carbon cloth. Scan rate: 50 mV·s^−1^.

The cyclic voltammograms (CVs) obtained in an acidic electrolyte without vanadium ions are shown in Figure 4. The current in the voltammogram is composed from three components, i.e., the electrochemical double-layer charging current at the carbon–electrolyte interface, and the faradaic currents due to the redox reactions of the surface functional groups and the carbon surface oxidation. The electrochemical double-layer charging yields a constant current and a rectangular CV shape. The current depends on the extent of the exposure of the basal and edge planes, the specific capacitances of which are 16 and 50–70 μF·cm^−2^ (microscopic actual surface area), respectively, according to the report by Yeager and co-workers [28]. The broad redox peaks around 0.5 V and the oxidation current above 0.8 V were attributed to the redox reactions of the quinone/hydroquinone-like surface functional groups and the carbon surface oxidation, respectively. These currents increased after the thermal oxidation and also increase with increasing temperature during thermal oxidation of TGP-CSnPc. The increase in the electrochemical double-layer current was attributed to the exposure of the edge planes, which is in agreement with the Raman spectra. The large carbon surface oxidation current observed for TGP-CSnPc-650Air implied the development of high surface roughness.

### Redox reactions of vanadium ions

**Figure 5:**
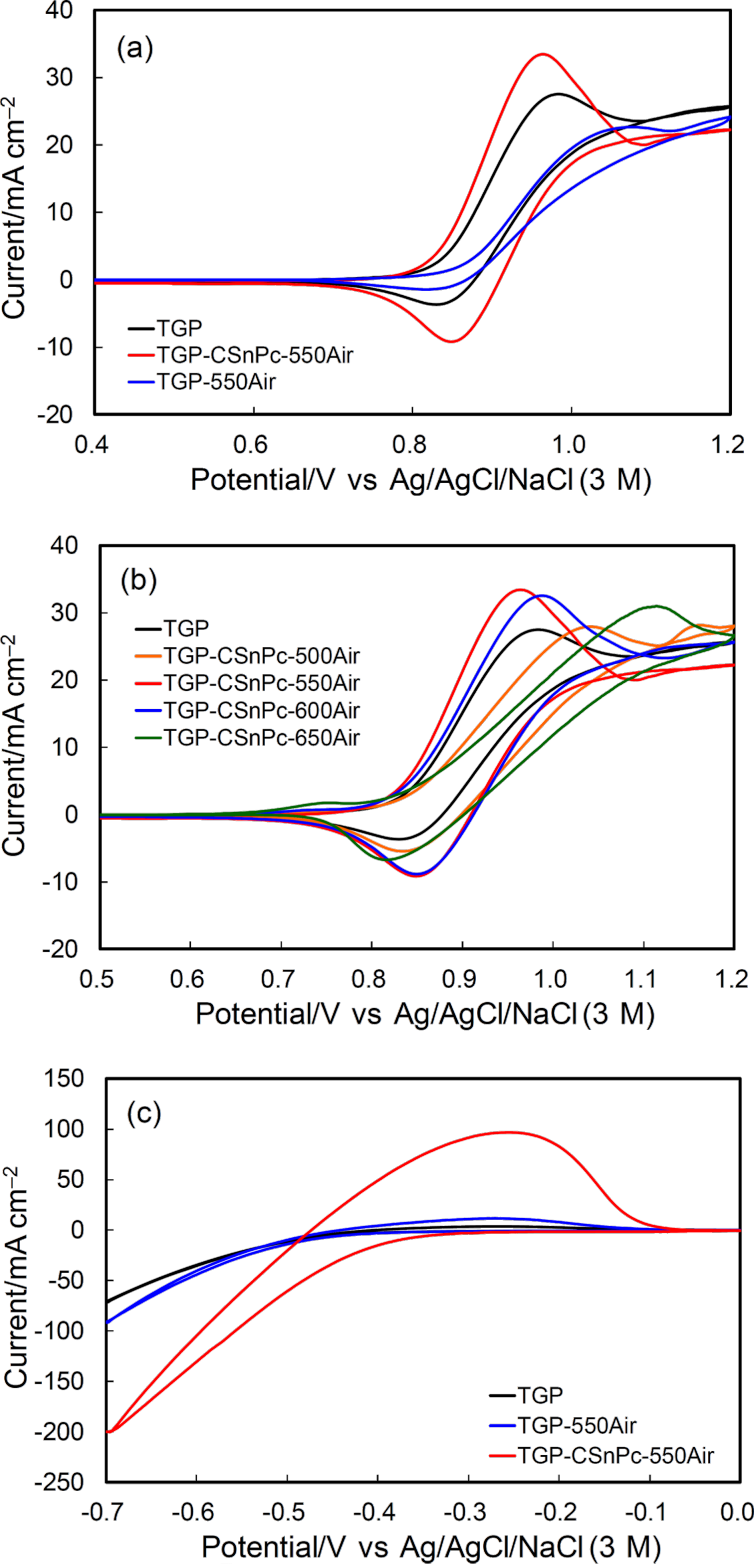
Cyclic voltammograms in 1 M VOSO_4_ + 2 M H_2_SO_4_ at 25 °C for (a,b) VO^2+^/VO_2_^+^, (c) V^2+/3+^ redox reactions at TGP, TGP-550Air, TGP-CSnPc-*T*Air (*T* =500, 550, 600 and 650 °C). The reference electrode was Ag/AgCl/NaCl (3 M). The counter electrode was carbon cloth. Scan rates were (a,b) 1 mV·s^−1^ and (c) 50 mV·s^−1^.

The CVs in the potential ranges corresponding to the positive and negative electrode reactions in an acidic electrolyte containing vanadium ions are shown in Figure 5 for TGP and TGP-CSnPc-*T*Air. The CVs for TGP-550Air are also shown for comparison. The enhancement of the VO^2+^/VO_2_^+^ redox reactions at the TGP-CSnPc-550Air electrode is clearly demonstrated by the negative and positive peak shifts for the oxidation and reduction peaks, respectively, as well as the increased peak currents. This enhancement was attributed to the loading with SnO_2_ nanoparticles, considering the limited increase in the activity by the exposure of the edge plane obtained in a previous study [15] without SnO_2_, and the lower activity for the only thermally oxidized surface (TGP-550Air), which suffered from inhibition by the adsorption of VO_2_ [23,29]. The excessive exposure of the edge plane led to an activity decrease at TGP-CSnPc-600Air and TGP-CSnPc-650Air due to this inhibition and the optimized activity was attained with TGP-CSnPc-550Air in this study. The drastic change in the surface structure was attributed to a temperature-dependent catalytic effect of the tin-oxide nanoparticles on the carbon surface oxidation leading to fine etching.

A significant enhancement of the activity for the V^2+/3+^ redox reactions was also observed at TGP-CSnPc-550Air. The activity was equivalent to that obtained for the finely etched surface obtained in the previous study [15] without SnO_2_ nanoparticles. Because the V^2+^ ions generated by the negative scan could be easily oxidized by VO^2+^ to generate V^3+^, a scan rate of 50 mV·s^−1^ was chosen in order to observe the V^2+^ oxidation current before its loss. Clear V^2+/3+^ redox peaks were absent due to the distortion of the voltammograms. Nevertheless, the information about the order of the activity of the electrodes (TGP < TGP-550Air << TGP-CSnPc-550Air) was satisfactory.

### Flow cell tests

**Figure 6:**
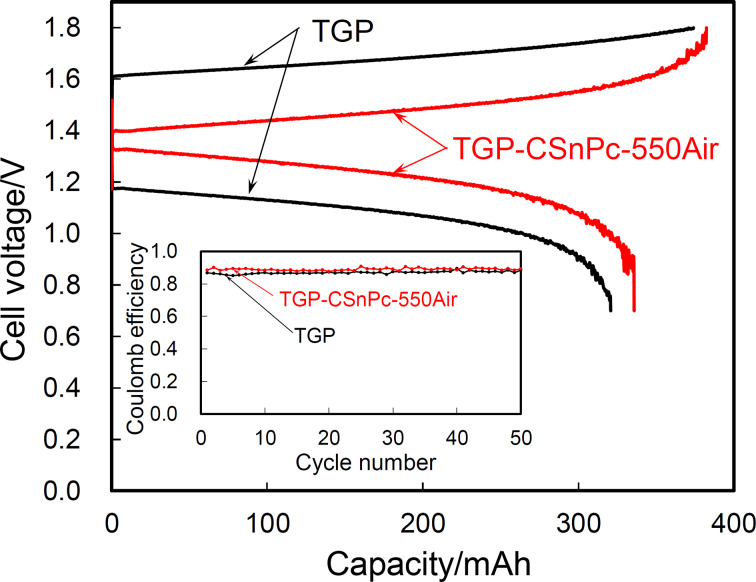
Charge–discharge curves and cycling performance for flow cells using three layers of TGP and TGP-CSnPc-550Air as electrodes. The electrode area was 3 cm^2^. The current density was 50 mA·cm^−2^. The flow rate was 3 cm^3^·min^−1^. The concentrations of the vanadium species and sulfate ion in the anolyte (20 cm^3^) and catholyte (20 cm^3^) were 1 and 3 M, respectively.

The flow cell was assembled using TGP or TGP-CSnPc-550Air in both the positive and negative electrodes to verify the enhancement effect observed by using cyclic voltammetry. Figure 6 shows the charge–discharge curves and cycling performances for the two full cells. Significant decreases in the overpotential for both charge and discharge processes were attained in the full cell with the TGP-CSnPc-550Air electrodes compared to that with the TGP electrodes. A stable coulomb efficiency is demonstrated by the cycling performance, indicating no influence of a potential Sn^2+^ contamination on the cycling performance. The finely etched surface and the slightly retained amorphous carbon might prevent potential dislocation and dissolution of the SnO_2_ particles.

## Conclusion

The thermal oxidation of Sn-containing carbonaceous thin films on a carbon fiber surface, which was formed by sublimation, deposition, and pyrolysis of SnPc during a single-step heat treatment in Ar atmosphere at 700 °C, achieved concurrent nanoscale surface etching and SnO_2_ loading on the carbon fibers. Both the positive and the negative electrode reactions of VRFB were enhanced and the full cell tests showed the significant decreases in the overpotential for both the charge and the discharge processes, as well as a stable cycling performance. A facile and efficient technique based on the nanoscale processing of the carbon fiber surface was presented to substantially improve the VRFB performance.

## Experimental

### Materials

Graphitic carbon paper (TGP-H-090, Toray, abbreviated as TGP), tin phthalocyanine (SnPc, Sigma-Aldrich), and ethanol (99.5%, Nacalai Tesque) were used as received. High-purity water was obtained by circulating ion-exchanged water through an Easypure water-purification system (Barnstead, D7403). Sulfuric acid (6 M, Kishida Chemical Co., Ltd.) was diluted with the high-purity water to prepare a 2 M H_2_SO_4_ solution. Oxovanadium sulfate hydrate, VOSO_4_·*n*H_2_O, was purchased from Sigma-Aldrich (purity > 99.99%) and Nacalai Tesque, which was dissolved in 2 M H_2_SO_4_ to prepare VOSO_4_ (1 M)/H_2_SO_4_ (2 M). The number of water of hydration, *n*, was provided by the manufacturer or determined in advance by thermogravimetry and a differential thermal analysis using an SSC/5200 thermal analyzer (Seiko Instruments).

### Concurrent surface etching and SnO_2_ loading

For depositing the Sn-containing carbonaceous thin films (CSnPc), eight pieces of TGP (1 cm^2^) and 10 mg of SnPc were placed in a crucible (15 cm^3^) with a cap and heat-treated at 700 °C for 1 h after raising the temperature at 5 °C·min^−1^ in an Ar atmosphere. The sample was labeled TGP-CSnPc. The heat treatment in air was performed for TGP-CSnPc at *T* = 500, 550, 600 and 650 °C for 1 h. The obtained samples were labeled TGP-CSnPc-*T*Air. For comparison, the heat treatment of TGP without CSnPc was also performed in air at 550 °C for 1 h (TGP-550Air).

### Characterization of carbon fiber surface

A field-emission scanning electron microscope (FESEM, JSM-6700F, JEOL) was used to observe the surface structure. The Raman spectra were obtained in backscattering mode by an NRS-3100 spectrometer (JASCO) using an Ar^+^-ion laser (532.05 nm, 0.3 mW) as the excitation source. The laser beam was focused on the surface of the carbon thin film, producing a spot (analysis area) of approximately 4 mm in diameter. A custom-written software using Microsoft Excel based on Gaussian functions was used for the Raman peak deconvolution and fitting. Energy-dispersive X-ray spectrometry was performed using a FESEM (JSM-7800F, JEOL) and EDX (Octane Elect Super, EDAX). X-ray photoelectron spectroscopy (XPS) was carried out using an AXIS ULTRA DLD system (Kratos Analytical) with Al Ka radiation (1486.6 eV) and the accompanying Vision processing software. The XPS analysis area was 0.3 × 0.7 mm.

### Electrochemical measurements

As described in [15], cyclic voltammetry was carried out using a three-electrode glass cell and an electrochemical analyzer, 100B/W (BAS). An Au wire as a lead was connected to the upper side of the 1 cm^2^ sample to form the working electrode. The electrode was immersed in ethanol and then rinsed with high-purity water to fully wet the electrode and to minimize the influence of wetting [7,30]. The counter electrode was carbon cloth (ElectroChem). The reference electrode was Ag/AgCl/NaCl (3 M) (0.212 V vs standard hydrogen electrode). The electrolytes were H_2_SO_4_ (2 M) and VOSO_4_ (1 M)/H_2_SO_4_ (2 M). The measurements were carried out under Ar atmosphere at 25 °C. A flow cell test was performed using three layers of 3 cm^2^ of TGP-CSnPc-550Air as the negative and positive electrodes, and Nafion 212 as the separator, incorporated into a flow cell similar to that used in a previous study [31]. The number of the layers was also chosen according to the results of this study. TGP-CSnPc-550Air was immersed in ethanol and rinsed with high-purity water before the incorporation. The anolyte (40 cm^3^) and catholyte (20 cm^3^) were prepared by electrolysis (charging) of 1 M VOSO_4_ + 2 M H_2_SO_4_ until the full conversion of VO^2+^ to VO_2_^+^ and V^2+^. After the electrolysis, half of the anolyte was removed and the pre-discharge was carried out at 50 mA·cm^−2^, followed by measurement of the charge–discharge curve. The flow rate was 3 cm^3^·min^−1^. The current density was 50 mA·cm^−2^. A flow cell using TGP was similarly tested for comparison.

## Supporting Information

File 1 Enlarged views of FESEM images, Raman spectra, C 1s XPS spectra, deconvoluted O 1s XPS spectra, and the content of surface oxygen species.
