# Supplementary material for: Concurrent nanoscale surface etching and SnO2 loading of carbon fibers for vanadium ion redox enhancement
Source: Beilstein J Nanotechnol. 2019 Apr 30;10:985–92. doi: 10.3762/bjnano.10.99 (PMC6541317; doi:10.3762/bjnano.10.99)
Supplement: File 1 — Enlarged views of FESEM images, Raman spectra, C 1s XPS spectra, deconvoluted O 1s XPS spectra, and the content of surface oxygen species. [file Beilstein_J_Nanotechnol-10-985-s001.pdf]

BEILSTEIN JOURNAL OF NANOTECHNOLOGY

Supporting Information

for

Concurrent nanoscale surface etching and SnO_2 loading of carbon fibers for vanadium ion redox enhancement

Jun Maruyama, Shohei Maruyama, Tomoko Fukuhara, Toru Nagaoka and Kei Hanafusa

Beilstein J. Nanotechnol. **2019,** *10,* 985–992. doi:10.3762/bjnano.10.99

Enlarged views of FESEM images, Raman spectra, C 1s XPS spectra, deconvoluted O 1s XPS spectra, and the content of surface oxygen species

License and Terms: This is a supporting information file under the terms of the Creative Commons Attribution License (http://creativecommons.org/licenses/by/4.0). Please note that the reuse, redistribution and reproduction in particular requires that the authors and source are credited.
The license is subject to the *Beilstein Journal of Nanotechnology* terms and conditions: (https://www.beilstein-journals.org/bjnano)

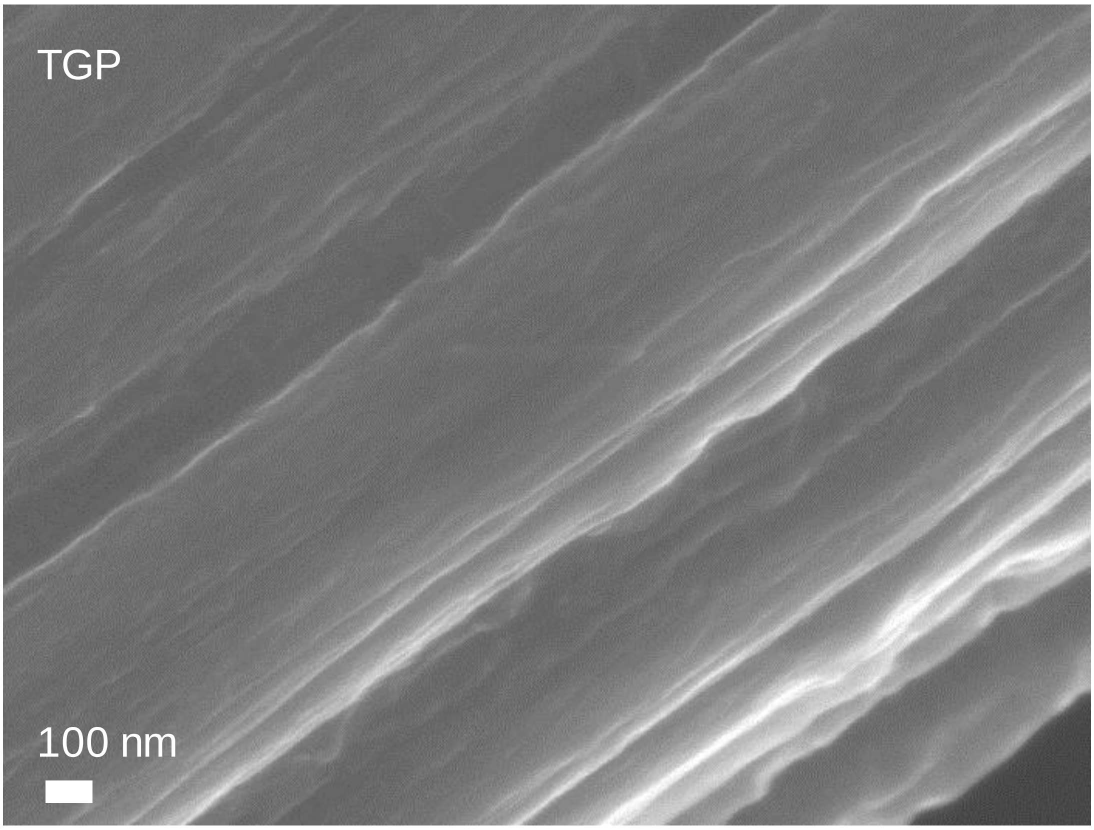

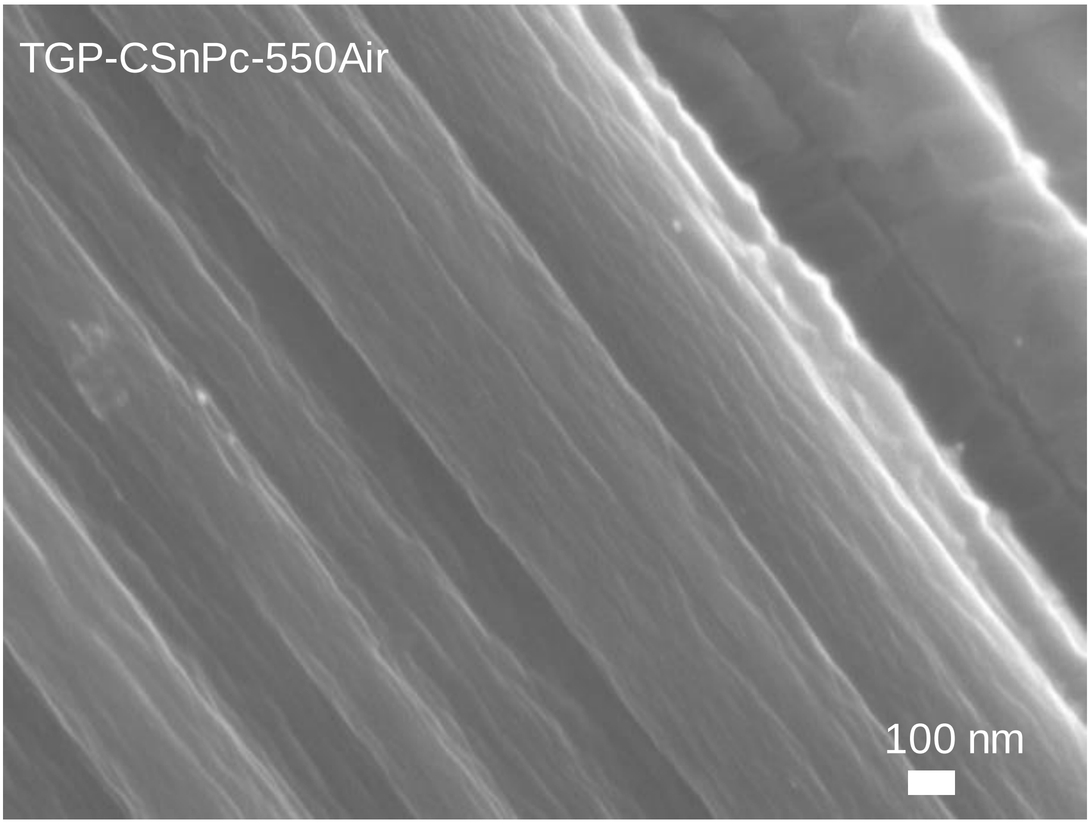

Figure S1: Enlarged views of FESEM images for TGP and TGP-CSnPc-550Air.

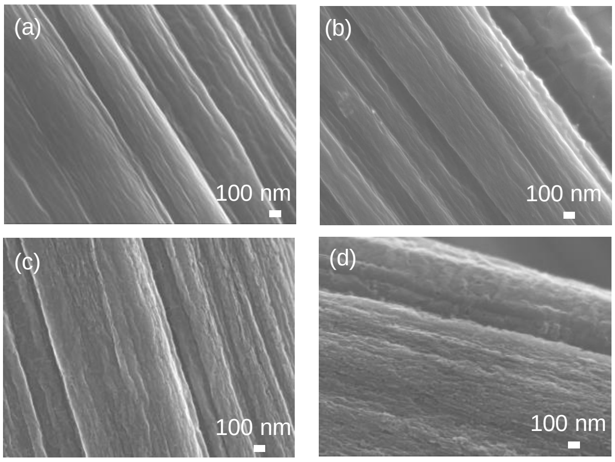

Figure S2: FESEM images of TGP-CSnPc-*T*Air (*T* = 500 (a), 550 (b), 600 (c), 650 °C (d)).

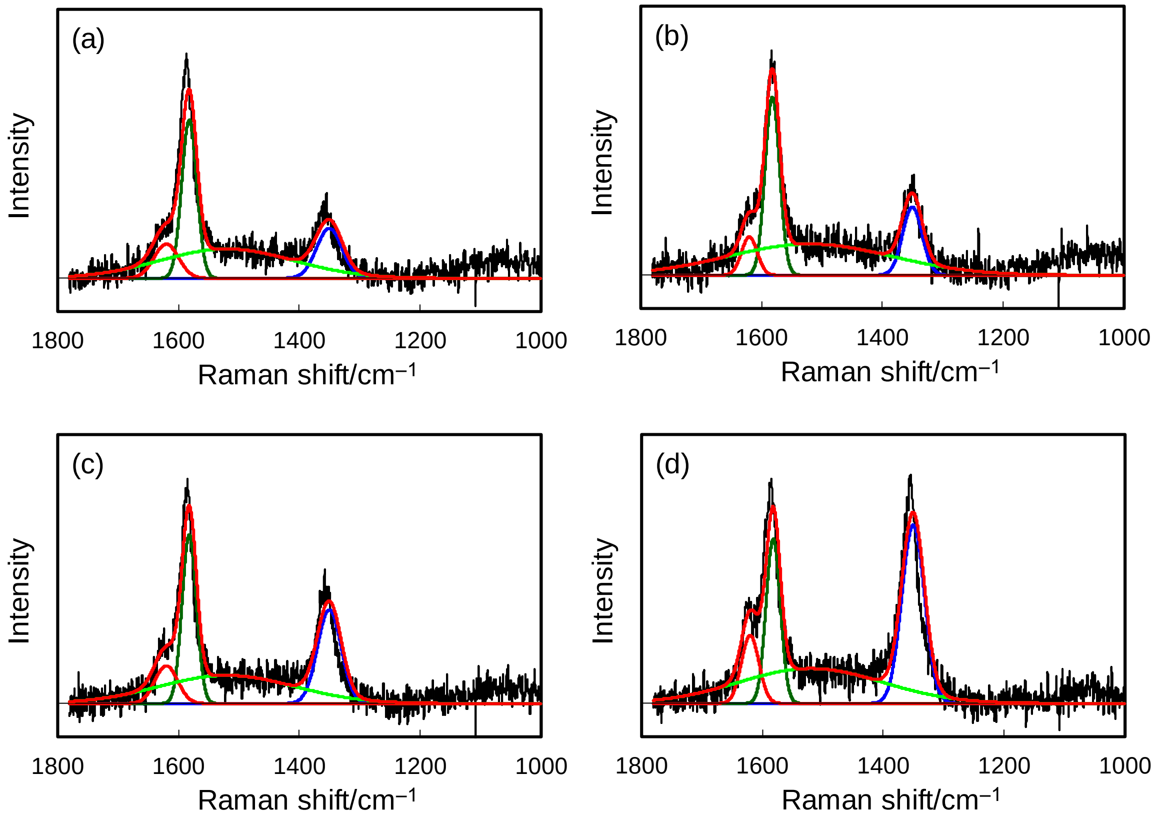

Figure S3: Raman spectra of TGP-CSnPc-*T*Air (*T* = 500 (a), 550 (b), 600 (c), 650 °C (d)).

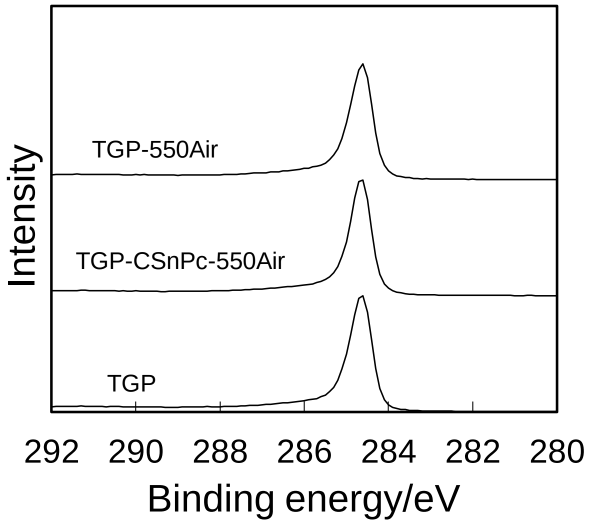

Figure S4: XPS spectra of C 1s in TGP, TGP-CSnPc-550Air, and TGP-550Air.

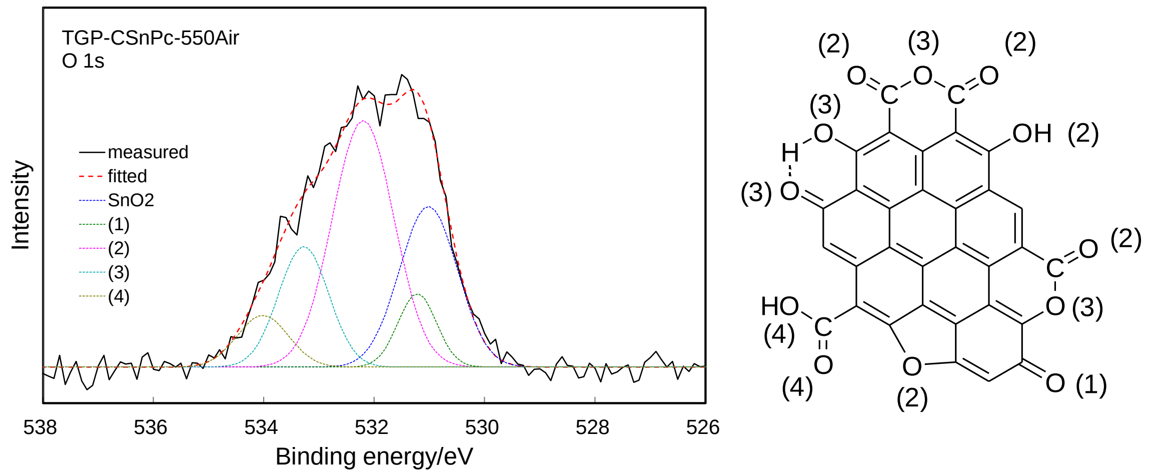

Figure S5: (a) X-ray photoelectron spectra of O 1s in TGP-CSnPc-550Air and possible bonding modes according to [1-4]; (b) scheme of O-containing functional groups cited from [1]. The numbers in parentheses in (a) correspond to the bonding modes represented in (b).

Table S1: Content of O species.

species	content [%]
SnO_2	25.9
mode (1)	7.9
mode (2)	42.2
mode (3)	16.8
mode (4)	7.3

References

1. Zielke, U.; Hüttinger, K. J.; Hoffman, W. P. *Carbon* **1996,** *34*, 983–998. doi:10.1016/0008-6223(96)00032-2
2. Badrinarayanan, S.; Mandale, A. B.; Gunjikar, V. G.; Sinha, A. P. B. *J. Mater. Sci.* **1986,** *21*, 3333–3338. doi:10.1007/BF00553376
3. Giulio, M. D.; Micocci, G.; Serra, A.; Tepore, A.; Rella, R.; Siciliano, P. *Sens. Actuators, B* **1995,** 24–25, 465-468. doi:10.1016/0925-4005(94)01397-7
4. Choi, W.-K.; Jung, H.-J.; Koh, S.-K. *J. Vac. Sci. Technol., A* **1996,** *14*, 359–366. doi:10.1116/1.579901
